# Supplementary material for: The impact of wild-boar-derived microbiota transplantation on piglet microbiota, metabolite profile, and gut proinflammatory cytokine production differs from sow-derived microbiota
Source: Appl Environ Microbiol. 2025 Feb 4;91(3):e02265-24. doi: 10.1128/aem.02265-24 (PMC11921332; doi:10.1128/aem.02265-24)
Supplement: Graphical abstract — Pictorial diagram of the study workflow. [file aem.02265-24-s0001.pdf]

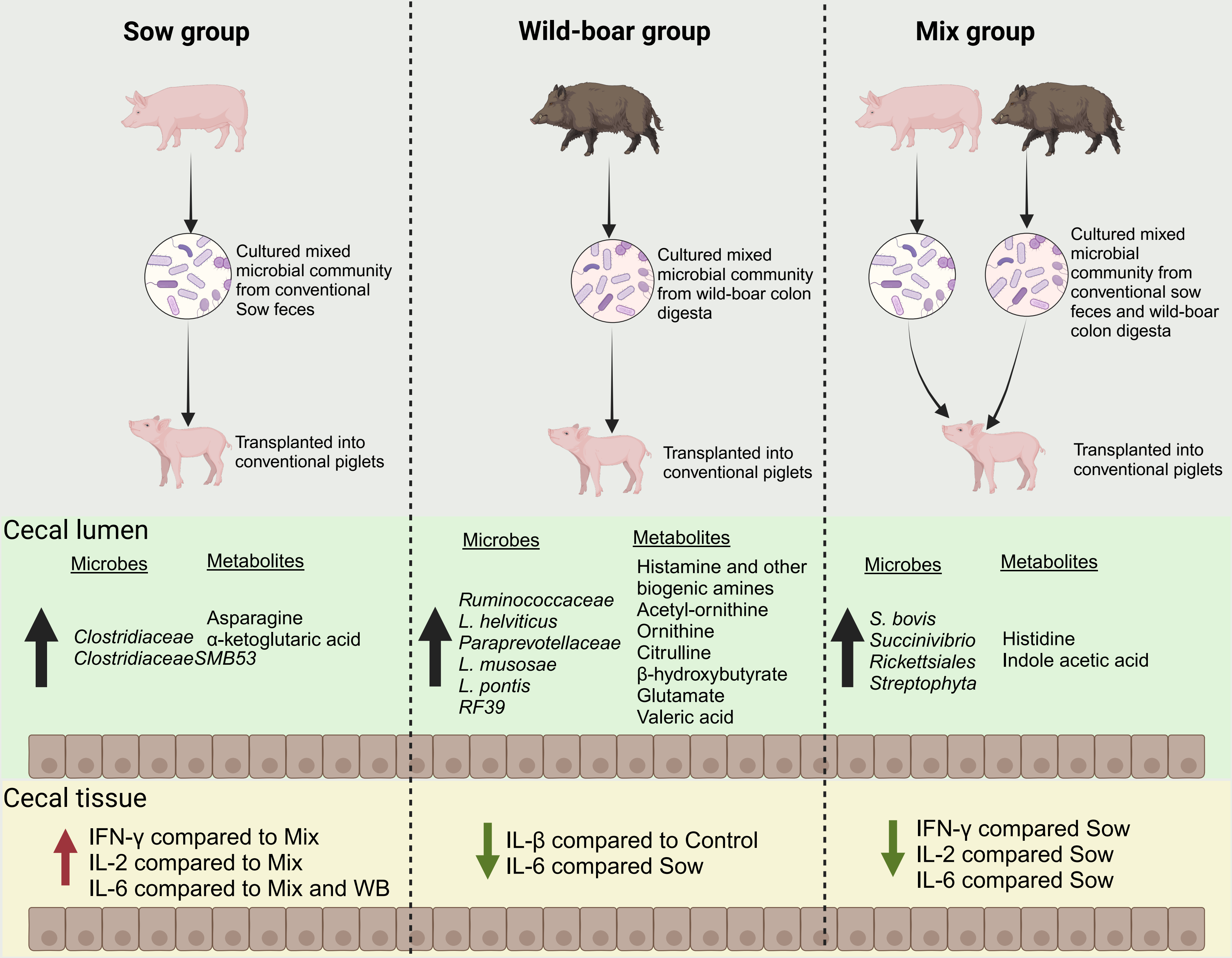

Sow group
Wild-boar group
Mix group
Cultured mixed microbial community from conventional Sow feces
Cultured mixed microbial community from wild-boar colon digesta
Cultured mixed microbial community from conventional sow feces and wild-boar colon digesta
Transplanted into conventional piglets
Transplanted into conventional piglets
Transplanted into conventional piglets
Cecal lumen
Microbes
Metabolites
Clostridiaceae
ClostridiaceaeSMB53
Asparagine
α-ketoglutaric acid
Microbes
Metabolites
Ruminococcaceae
L. helviticus
Paraprevotellaceae
L. musosae
L. pontis
RF39
Histamine and other biogenic amines
Acetyl-ornithine
Ornithine
Citrulline
β-hydroxybutyrate
Glutamate
Valeric acid
Microbes
Metabolites
S. bovis
Succinivibrio
Rickettsiales
Streptophyta
Histidine
Indole acetic acid
Cecal tissue
IFN-γ compared to Mix
IL-2 compared to Mix
IL-6 compared to Mix and WB
IL-β compared to Control
IL-6 compared Sow
IFN-γ compared Sow
IL-2 compared Sow
IL-6 compared Sow
