# Supplementary material for: The impact of wild-boar-derived microbiota transplantation on piglet microbiota, metabolite profile, and gut proinflammatory cytokine production differs from sow-derived microbiota
Source: Appl Environ Microbiol. 2025 Feb 4;91(3):e02265-24. doi: 10.1128/aem.02265-24 (PMC11921332; doi:10.1128/aem.02265-24)
Supplement: Supplemental material — Table S1 and Figures S1 to S5. [file aem.02265-24-s0002.pdf]

Supplementary Table 1: List of 70 metabolites in TMIC prime assay.

Metabolite class	*Metabolite*
Amine Oxide	Trimethylamine N-oxide
Amino Acids	Alanine, Arginine, Asparagine, Aspartate, Citrulline, Glutamine, Glutamate, Glycine, Histidine, Isoleucine, Leucine, Lysine, Methionine, Ornithine, Phenylalanine, Proline, Serine, Threonine, Tryptophan,Tyrosine, Valine, Betaine
Amino Acid Derivatives	Creatine, Phosphocreatine, Methylhistidine
Biogenic Amines	Acetyl-ornithine, ADMA, Asymmetric dimethylarginine, Total dimethylarginine, Alpha-Aminoadipic acid, Carnosine, Creatinine, Dihydroxyphenylalanine, Dopamine, Histamine, Kynurenine, Methioninesulfoxide, Hydroxyproline, Hydroxyproline, Nitrotyrosine, Phenylethylamine, Putrescine, Sarcosine, Serotonin, Spermidine, Spermine, Diacetylspermine, Taurine, Tyramine
Monosaccharides	Glucose
Organic Acids	Lactate, Beta-hydroxybutyric acid, Alpha-ketoglutarate, Citric acid, Butyrate, Propionic acid, Valeric acid, Para-hydroxyhippuric acid, Succinic acid, Fumaric acid, Pyruvic acid, Isobutyric acid, Hippuric acid, Methylmalonic acid, Acetic acid, Indole acetic acid, Uric acid
Vitamins & Cofactors	Choline

ADMA: Asymmetric dimethylarginine

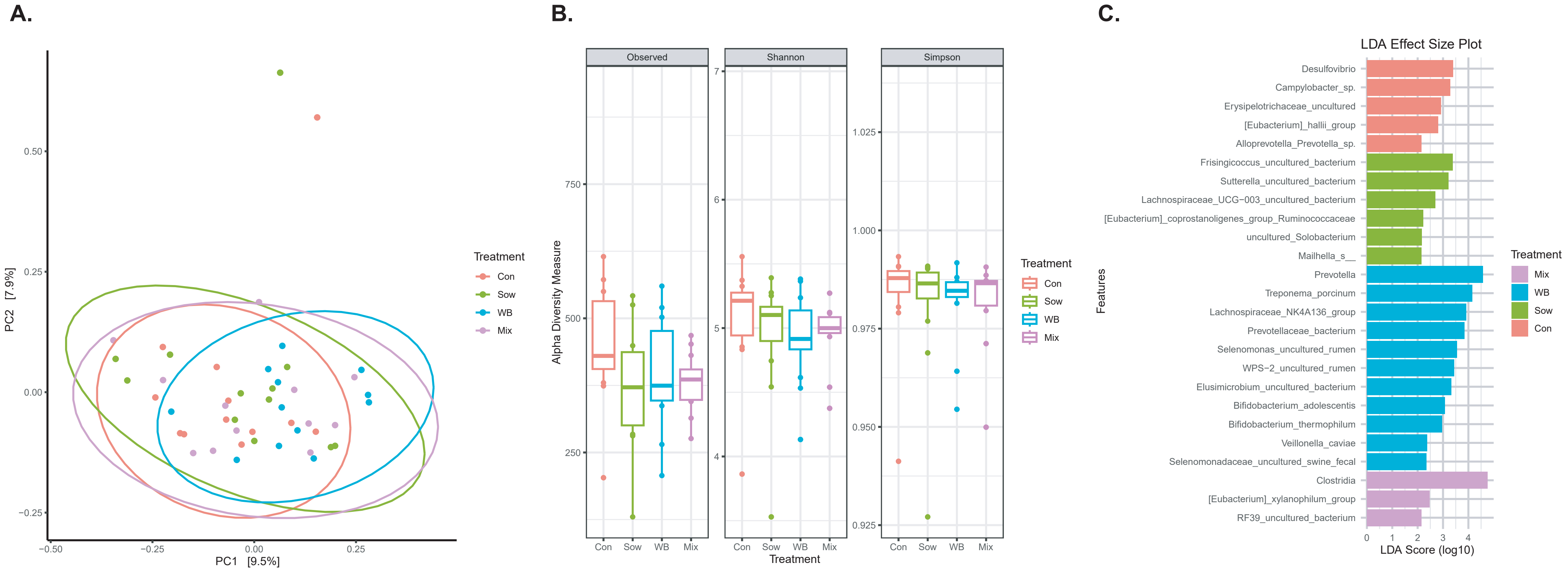

Supplementary Figure 1. Comparison of post-transplantation day 6 (PND 27) fecal microbiota composition and α-diversity among treatment groups (n = 12/group). (A) At day 6 (PND 27) post-transplantation, there was a trend of a shift in microbial community structure as measured by β-diversity based on Bray–Curtis dissimilarity (Adonis, $R^2 = 0.07$, $P = 0.07$; Betadispersion $P = 0.90$). (B) No differences were observed in α-diversity indices among treatment groups at day 6 (PND 27) post-transplantation (Observed $P = 0.18$; Shannon $P = 0.36$; Simpson $P = 0.38$). (C) LEfSe analysis identified differentially abundant taxonomical features among treatment groups ($P < 0.05$).

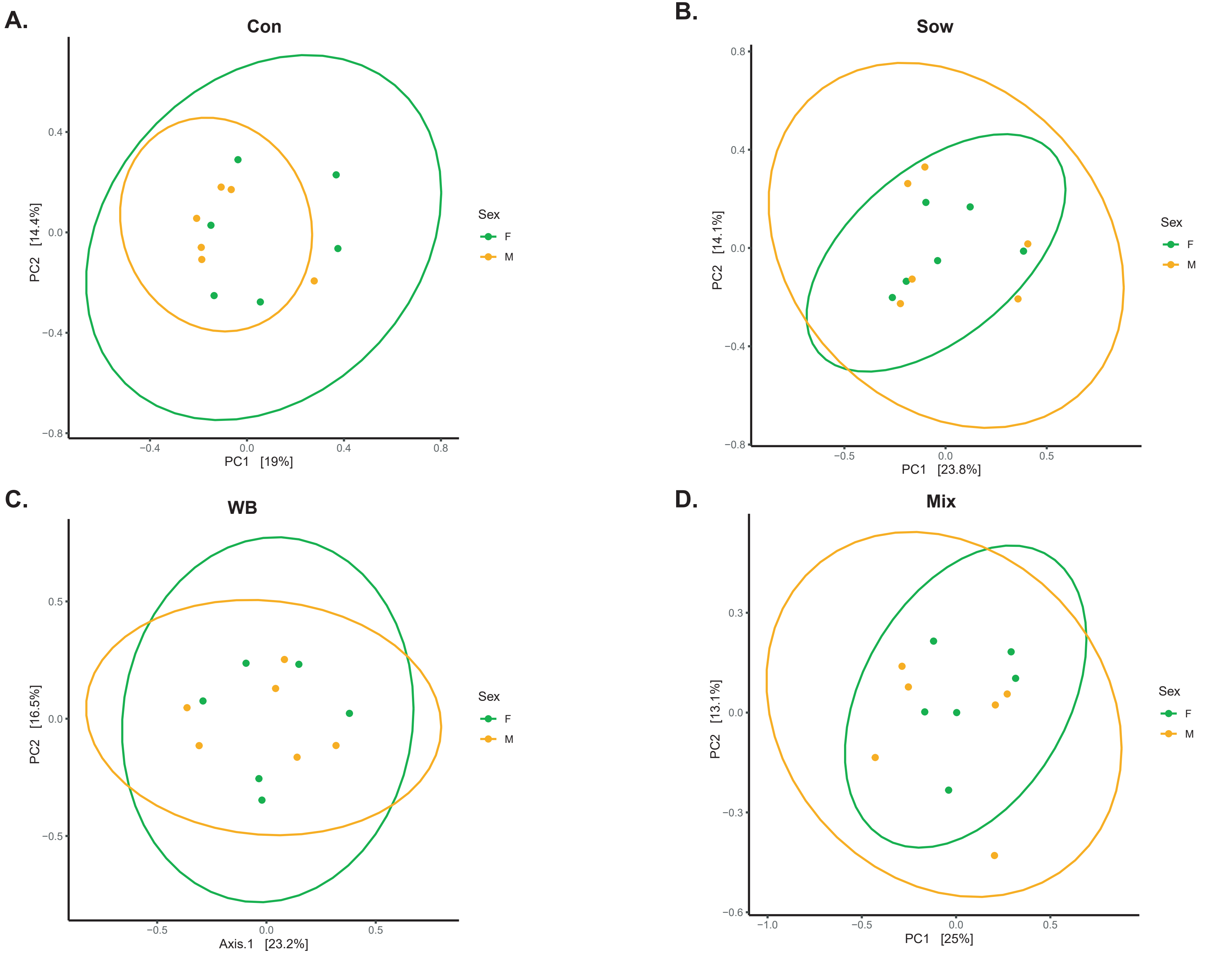

Supplementary Figure 2. Comparison of post-transplantation day 28 (PND 48) fecal microbial community structure based on sex showed no differences among treatment groups as measured by β-diversity based on Bray–Curtis dissimilarity. (A) Control (Adonis, $R^2 = 0.09$, $P = 0.47$; Betadispersion $P = 0.17$). (B) Sow (Adonis, $R^2 = 0.08$, $P = 0.57$; Betadispersion $P = 0.46$). (C) WB (Adonis, $R^2 = 0.07$, $P = 0.79$; Betadispersion P = 0.76). (D) Mix (Adonis, $R^2 = 0.08$, P = 0.62; Betadispersion $P = 0.06$). F: Female; M: Male.

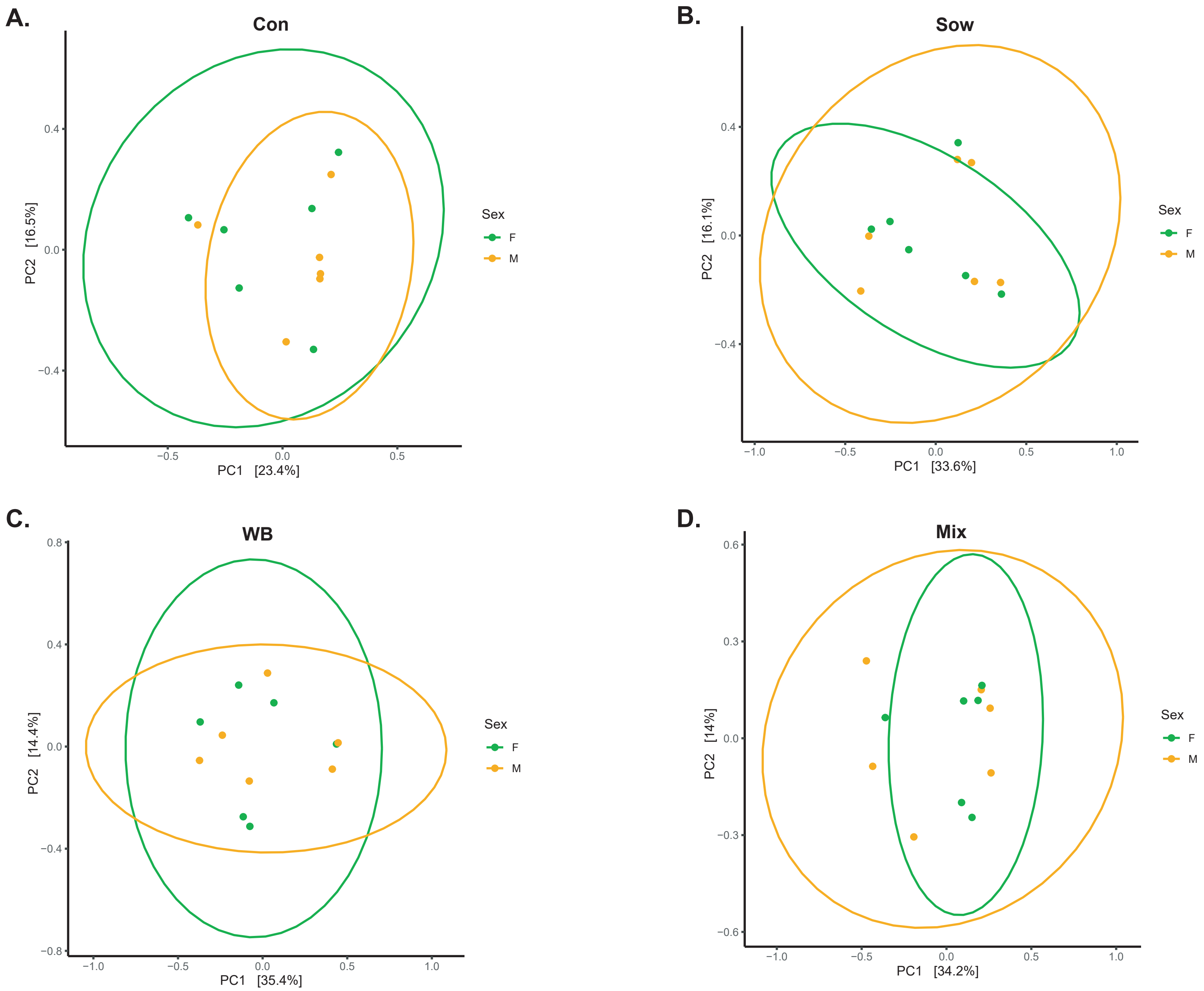

Supplementary Figure 3. Comparison of post-transplantation day 28 (PND 48) cecal microbial community structure based on sex showed no differences among treatment groups as measured by β-diversity based on Bray–Curtis dissimilarity. (A) Control (Adonis, $R^2 = 0.06$, $P = 0.93$; Betadispersion $P = 0.70$). (B) Sow (Adonis, $R^2 = 0.04$, $P = 0.99$; Betadispersion $P = 0.59$). (C) WB (Adonis, $R^2 = 0.05$, $P = 0.94$; Betadispersion $P = 0.29$). (D) Mix (Adonis, $R^2 = 0.07$, $P = 0.67$; Betadispersion $P = 0.16$). F: Female; M: Male.

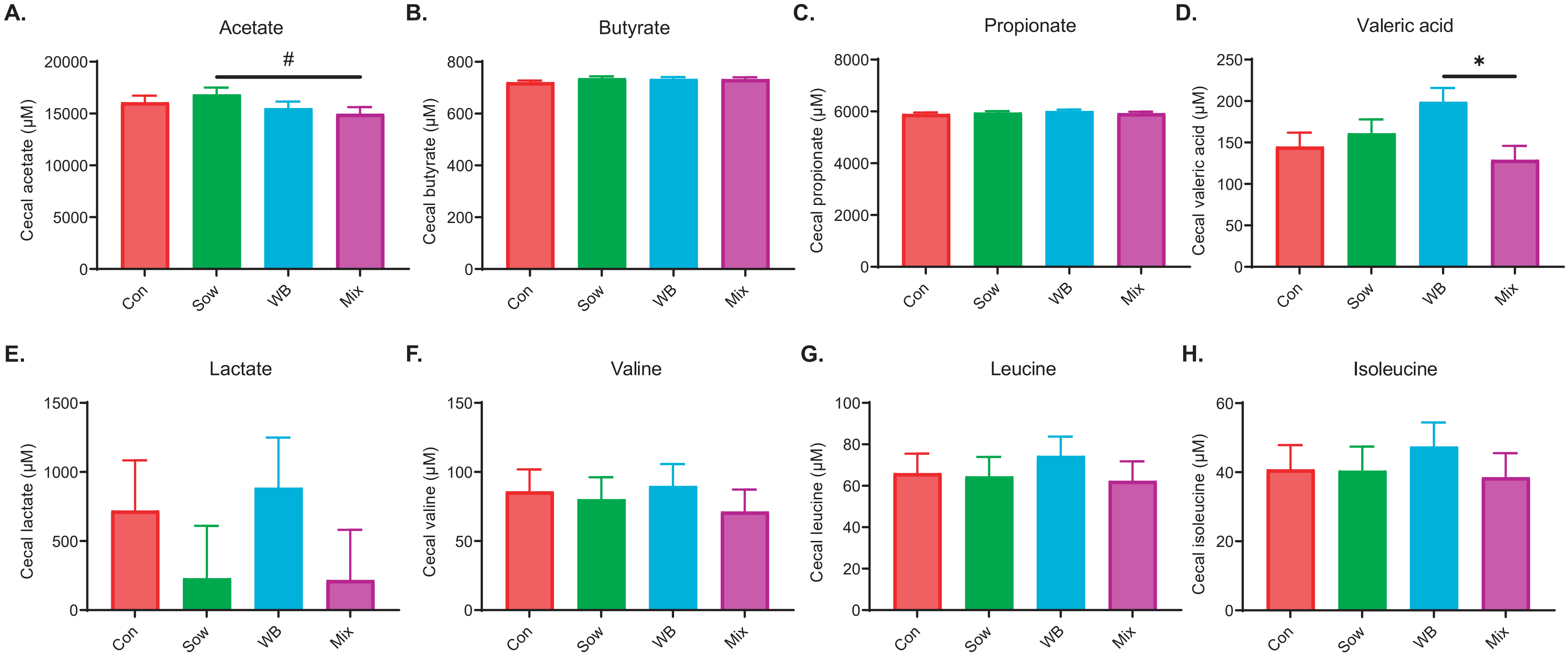

Supplementary Figure 4: Comparison of cecal SCFAs, lactate, and branch-chain amino acid concentrations among treatment groups. (A) Acetate; (B) Butyrate; (C) Propionate; (D) Valeric acid; (E) Lactate; (F) Valine; (G) Leucine; and (H) Isoleucine (n = 12/group). All data is shown as a mean with SEM. *P*-values were obtained from adjusted pairwise comparisons after a linear mixed effect model (where litter and pen effect accounted for a random effect) and significance was considered at $P < 0.05$. $^*P < 0.05$, $^{**}P < 0.01$, $^{***}P < 0.001$, # $P > 0.05$ and < 0.1

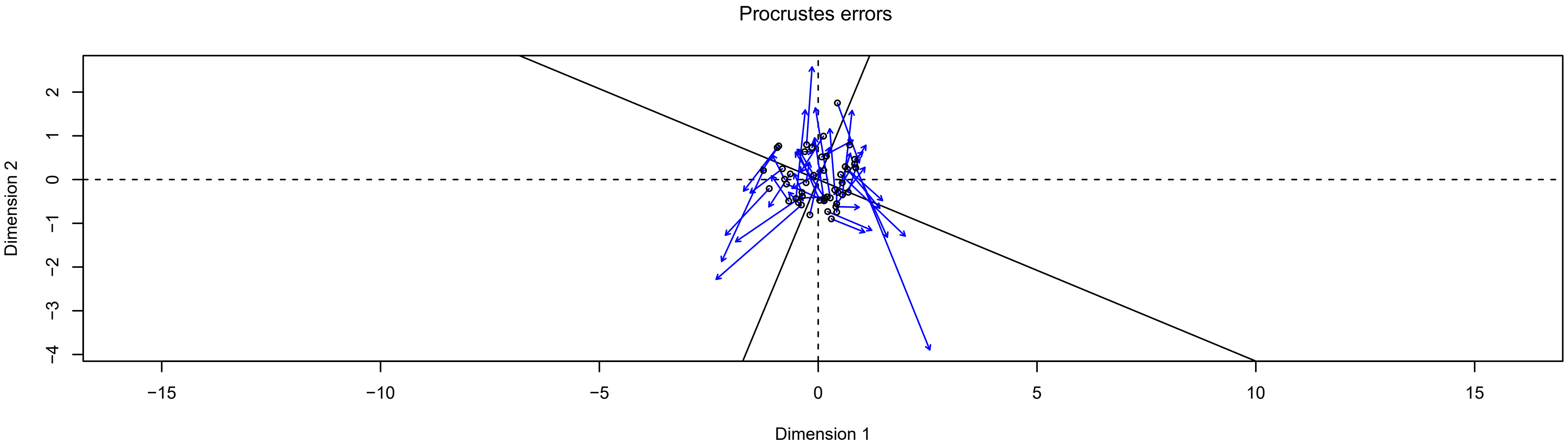

Supplementary Figure 5: Procrustes analysis on the principal-coordinate analysis axes of the cecal microbiome and metabolome. An association between cecal metabolites and microbiota compositions was observed (Procrustes sum of squares, 0.75; correlation, 0.49; $P < 0.001$). Greater distances between the black hollow circles representing metabolite eigenvalues and the blue arrows representing microbiota eigenvalues indicated a higher level of discordance between the datasets for each sample.
